# Estimation of vaccine efficacy in a repeated measures study under heterogeneity of exposure or susceptibility to infection

**DOI:** 10.1098/rsta.2008.0044

**Published:** 2008-04-11

**Authors:** Clarissa Valim, Maura Mezzetti, James Maguire, Margarita Urdaneta, David Wypij

**Affiliations:** 1 Clinical Research Program, Children's Hospital Boston300 Longwood Ave., Boston, MA 02115, USA; 2 Department of Pediatric Surgery, Harvard Medical SchoolMA 02115, USA; 3 Department of Biostatistics, Harvard School of Public HealthBoston, MA 02115, USA; 4 Department of Immunology and Infectious Diseases, Harvard School of Public HealthBoston, MA 02115, USA; 5 Faculty of Economics, Tor Vergata University00133 Rome, Italy; 6 Health Surveillance Bureau, Ministry of HealthBR 70.058-900, Brazil; 7 Department of Pediatrics, Harvard Medical SchoolBoston, MA 02115, USA

**Keywords:** vaccine, efficacy, generalized linear models, longitudinal analyses, generalized estimating equation, heterogeneity

## Abstract

Vaccine efficacy (VE) is commonly estimated through proportional hazards modelling of the time to first infection or disease, even when the event of interest can recur. These methods can result in biased estimates when VE is heterogeneous across levels of exposure and susceptibility in subjects. These two factors are important sources of unmeasured heterogeneity, since they vary within and across areas, and often cannot be individually quantified. We propose an estimator of VE per exposure that accounts for heterogeneous susceptibility and exposure for a repeated measures study with binary recurrent outcomes. The estimator requires only information about the probability distribution of environmental exposures. Through simulation studies, we compare the properties of this estimator with proportional hazards estimation under the heterogeneity of exposure. The methods are applied to a reanalysis of a malaria vaccine trial in Brazil.

## 1. Introduction

Vaccine efficacy (VE) is defined as the per cent reduction in the probability or hazard of disease conferred by the vaccine to an individual. It is typically estimated based on a marginal, or population-based, parameter, which is an average of the individual vaccine effects, specific to a geographically and temporally defined population (Halloran *et al*. 1991). Commonly, estimates of VE are based on one minus the hazard ratio from a proportional hazards model for time to first event, which can be an infection or disease, even when the disease under study recurs (Alonso *et al*. 1994; Bojang *et al*. 2001; Aponte *et al*. 2007). The use of the proportional hazards model in VE studies is widespread owing to its ease of implementation, interpretation and flexibility regarding the shape of the baseline hazard over time. An important advantage of proportional hazards models is that, in balanced randomized trials and under the proportional hazards assumption, the population VE represents the individual VE (Halloran *et al*. 1994; Gilbert *et al*. 1998), which is the causal parameter of interest. This individual VE in trials represents the experimental or biological effect of the vaccine and is useful in selecting or comparing the vaccine candidates. The population VE for the same vaccine could vary for different studies and does not allow an assessment of the efficacy of the vaccine itself.

Under homogeneity of risk factors of infection or disease in the population or of the VE itself, proportionality of the hazards may hold true (Vaupel *et al*. 1979; Aalen 1988; Hougaard 1995). However, the assumption of homogeneity is often unrealistic because fundamental risk factors for infectious diseases, such as individual exposure levels and susceptibilities to the pathogen pre-vaccination, are likely to be heterogeneous in the population (Struchiner *et al*. 1994; Halloran *et al*. 1995). In this paper we will focus on the heterogeneity due to exposure, assuming that the heterogeneous susceptibilities can be minimized in the design or analysis, e.g. by using covariates. Examples of covariates to model heterogeneous susceptibility include age or other markers of previous exposure history, such as time living in the area (Baird *et al*. 1993).

Exposure intensity varies greatly within and across populations and cannot always be reasonably and accurately quantified through covariates. The intensity of exposure can vary across individual behavioural characteristics such as occupation or sexual behaviour and is likely to fluctuate within geographical regions due to environmental factors (Smith *et al*. 1995). Under heterogeneity, proportional hazards analysis of time to first event can underestimate the individual VE, owing to unmeasured covariates representing prognostic factors for survival or random heterogeneity (Gail *et al*. 1984; Struthers & Kalbfleisch 1986; Schumacher *et al*. 1987; Aalen 1988, 1994; Chastang *et al*. 1988; Lin & Wei 1989; Omori & Johnson 1993; Schmoor & Schumacher 1997; Henderson & Oman 1999). Basically, unmeasured heterogeneity affects the comparability between vaccinees and non-vaccinees achieved by randomization in the beginning of the study. Intuitively, if the vaccine is effective, higher risk (more exposed) unvaccinated subjects fail faster than vaccinated subjects and are removed from the risk set.

Population heterogeneities in exposure intensities can be modelled using random effects survival methods, including Cox models with random effects (Hougaard 1995; Halloran *et al*. 1996; Longini & Halloran 1996). When infection or disease does not confer long-lasting immunity and may recur, multivariate survival methods may be used (Hougaard 2000; Therneau & Grambsch 2000). Multiple events may be analysed with Poisson regression or using a more flexible approach, such as the marginal model originally proposed by Anderson & Gill (1982) with variances adjusted for correlations within subjects using robust variance estimates (Lin & Wei 1989). Analyses of multiple events using the Anderson–Gill model are expected to correct for bias due to unmeasured heterogeneity because, since subjects remain in the risk set until the end of the study, vaccinated and unvaccinated subjects remain comparable. However, continuous time survival analysis methods typically require information about the exact failure times, though often in practice we can only distinguish a subject's outcomes in the time interval between consecutive visits (active case detection). Even when studies combine active case detection with passive case detection (detection of the event whenever people seek care), many of their cases are found through active detection and have event time interval censored. Under those circumstances, although the exact failure time could be approximated, a repeated measures analysis for a binary outcome represents a practical alternative to handle the sequential monitoring of subjects, interval-censored observations, recurrent episodes and non-proportional hazards.

In this paper, we present an estimator for VE given one exposure contact in a repeated measures analysis with a generalized estimating equations (GEE) approach, accounting for heterogeneous exposure and susceptibility pre-vaccination. Although the proposed estimator may be applied to different vaccines, we focus our model on estimating efficacy for malaria vaccines. Malaria is a leading cause of child mortality in developing countries (WHO 1995) and is transmitted by a mosquito vector. The transmission intensity of malaria is highly variable across and within regions, and highly seasonal (Smith *et al*. 1995). Immunity to malaria is partial and does not prevent recurrences of infection or disease. Currently there are several vaccines in the preclinical test phase (Aide *et al*. 2007). For some malaria vaccines, recent randomized trials have been performed in which the VE is primarily estimated based on the proportional hazards modelling of time to first event (Alonso *et al*. 2005; Bejon *et al*. 2006, 2007). It is not feasible to record exposure to infected mosquito bites received by each trial subject, and so exposure information typically relies on mosquito collections in samples of the study area. Case detection methods rely on both passive case detection, when individuals seek medical treatment, and active case detection, when houses are visited and individuals are examined at regular time intervals.

Section 2 presents our general model and estimator of VE. Under some simplifying assumptions, we propose estimation procedures using standard commercial software. In §3 we show that, with heterogeneity of exposure to infection, VE estimates based on the proportional hazards modelling of time to first event yield biased estimates of the individual VE. We also use simulation studies to compare our repeated measures estimator of VE with the proportional hazards model estimator of VE, when the assumption of proportional hazards holds. In §4, we apply the proposed model to a reanalysis of the data from a malaria SPf66 vaccine trial carried out in Brazil.

## 2. Model description

In a VE study, subjects *i*, for *i*=1, …, *n*, with different susceptibility status are randomized to vaccination (*V*_*i*_=1) or placebo (*V*_*i*_=0) at the beginning of the study period. The outcome status (*D*_*ij*_) of each subject (binary) is subsequently recorded at specific time intervals, *j*. For vaccines targeting recurrent events, after an event, a subject treated can re-enter the risk set and present the outcome again. The probability of presenting the outcome at each interval *j* depends on the specific amount of exposures received (*W*_*ij*_), which arrive with intensity *λ*_*ij*_. Although *W*_*ij*_ cannot be measured, *λ*_*ij*_ may be estimated in a separate ecological study or indirectly, allowing one to determine the empirical probability distribution of exposure. In this scenario, an estimator of VE can be defined based on the likelihood of

$$Pr\left\{d_{ij}|v_{i};\lambda_{ij}\right\}=\sum_{w_{ij}}Pr\left\{d_{ij}|v_{i},w_{ij}\right\}Pr\left\{w_{ij};\lambda_{ij}\right\},$$

where $Pr\left\{w_{ij};\lambda_{ij}\right\}$ is the probability distribution of exposure contacts for subject *i* in time interval *j*. Define a subject's susceptibility status, or the probability of presenting the outcome (baseline probability) in one exposure contact as $p_{ij}=f\left(\alpha_{ij}^{\text{T}}x_{ij}\right)$, where ***x***_*ij*_ are covariates, possibly time varying. Assuming that susceptibility to the vaccine is reduced by a multiplicative factor *g*(·), independent of the susceptibility *p*_*ij*_ across exposures, we can define the probability of presenting the outcome given *W*_*ij*_ exposures as

$$Pr\left\{d_{ij}|v_{i},w_{ij}\right\}=1-{\left[1-p_{ij}g(\cdot )\right]}^{w_{ij}}.$$

The reduction in the probability of disease given one exposure conferred by the vaccine can be defined as $g\left(\beta_{i}v_{i}+\delta_{ij}^{\text{T}}x_{ij}v_{i}\right)$, allowing the VE to vary across subjects and to interact with covariates.

Different functions can be chosen to model *f*, *g* and *λ* and different probability distributions to model $Pr\left\{w_{ij};\lambda_{ij}\right\}$. If *f* and *g* are exponential functions (log link model), we can write the expression for the probability of disease for the *i*th subject at the *j*th interval given vaccination status as

(2.1) $$Pr\left\{d_{ij}=1|v_{i},x_{ij};\alpha ,\beta_{i},\delta ,\lambda \right\}=\sum_{w_{ij}}\left\{1-{\left[1-\text{exp}\left(\alpha_{ij}^{\text{T}}x_{ij}+\beta_{i}v_{i}+\delta_{ij}^{\text{T}}x_{ij}v_{i}\right)\right]}^{w_{ij}}\right\}Pr\left\{w_{ij};\lambda_{ij}\right\}.$$

The resulting contribution to the likelihood of the *i*th subject at the *j*th time interval becomes

$$L_{ij}=\sum_{w_{ij}}{\left\{1-{\left[1-\text{exp}\left(\alpha_{ij}^{\text{T}}x_{ij}+\beta_{i}v_{i}+\delta_{ij}^{\text{T}}x_{ij}v_{i}\right)\right]}^{w_{ij}}\right\}}^{d_{ij}}{\left\{{\left[1-\text{exp}\left(\alpha_{ij}^{\text{T}}x_{ij}+\beta_{i}v_{i}+\delta_{ij}^{\text{T}}x_{ij}v_{i}\right)\right]}^{w_{ij}}\right\}}^{1-d_{ij}}Pr\left\{w_{ij};\lambda_{ij}\right\}.$$

Several simplifying assumptions can be made. For instance, if we let *W*_*ij*_ be independently Poisson (*λ*_*ij*_) distributed and $s=1-\;\text{exp}\left(\alpha_{ij}^{\text{T}}x_{ij}+\beta_{i}v_{i}+\delta_{ij}^{\text{T}}x_{ij}v_{i}\right)$, then

$$Pr\left\{d_{ij}=1|v_{i},x_{ij};\alpha ,\beta ,\delta ,\lambda \right\}=1-E_{W}\left[s^{W}\right],$$

where $E_{W}\left[s^{W}\right]$ is the generating function of a Poisson random variable. Algebraic manipulation reduces equation (2.1) to

$$\ln\left(-\ln\left(1-Pr\left\{d_{ij}=1|v_{i},x_{ij};\alpha ,\beta_{i},\delta ,\lambda \right\}\right)\right)=\log(\lambda_{ij})+\alpha_{ij}^{\text{T}}x_{ij}+\beta_{i}v_{i}+\delta_{ij}^{\text{T}}x_{ij}v_{i}.$$

Thus, the probability of disease for the *i*th subject at the *j*th interval is linked to the parameters by a complementary log–log link function, allowing the estimation of VE through standard statistical software. In this model, the probability distribution of exposure contacts may also be allowed to vary over time and across covariate levels by $\lambda_{ij}=\lambda \left(\gamma_{ij}^{\text{T}}z_{ij}\right)$. For instance, in the case of the malaria vaccine, the intensity of exposure can be estimated based on an ecological study of the number of infectious bites received by subjects at different months (or seasons) of the year and whether or not the subject uses a bed net.

In the above models, the individual VE or VE in one exposure contact varies across subjects and intervals and can be expressed by

$$VE_{W_{ij}=1}=1-\frac{Pr\left\{d_{ij}=1|v_{i}=1,w_{ij}=1,x_{ij};\alpha ,\beta ,\delta ,\lambda \right\}}{Pr\left\{d_{ij}=1|v_{i}=0,w_{ij}=1,x_{ij};\alpha ,\beta ,\delta ,\lambda \right\}}=1-\exp\left(\beta +\delta_{ij}^{\text{T}}x_{ij}\right).$$

In the absence of interaction between the vaccine and covariates (*δ*=0), the VE given one exposure contact is homogeneous across subjects, even when the baseline probability of an event or susceptibility in one exposure contact is varying. VE varies in the range (0, 1] when the vaccine is beneficial.

The population VE or VE marginal on exposure can be expressed as

$$VE=1-\frac{Pr\left\{d_{ij}=1|v_{i}=1,x_{ij};\alpha ,\beta ,\delta ,\lambda \right\}}{Pr\left\{d_{ij}=1|v_{i}=0,x_{ij};\alpha ,\beta ,\delta ,\lambda \right\}}=1-\frac{\sum_{w_{ij}}Pr\left\{d_{ij}=1|v_{i}=1,x_{ij};\alpha ,\beta ,\delta ,\lambda \right\}Pr\left\{w_{ij};\lambda_{ij}\right\}}{\sum_{w_{ij}}Pr\left\{d_{ij}=1|v_{i}=0,x_{ij};\alpha ,\beta ,\delta ,\lambda \right\}Pr\left\{w_{ij};\lambda_{ij}\right\}}=1-\frac{1-\;\text{exp}\left\{-\lambda_{ij}\;\text{exp}\left\{\alpha_{ij}^{\text{T}}x_{ij}+\beta_{i}+\delta_{ij}^{\text{T}}x_{ij}\right\}\right\}}{1-\;\text{exp}\left\{-\lambda_{ij}\;\text{exp}\left\{\alpha_{ij}^{\text{T}}x_{ij}\right\}\right\}}.$$

Straightforward extensions of this model could involve random effects or Markov covariates (Diggle *et al*. 1994). The appropriateness of each of these approaches is related to the existence of heterogeneity other than that caused by exposure, i.e. heterogeneity in susceptibility not measured by the modelled covariates and heterogeneity in the VE per exposure. Alternative approaches will lead to different interpretation of the VE in one exposure.

Throughout this paper, we will model the marginal probability of disease given exposure. The marginal models assume that event history during the study period does not affect the susceptibility per exposure. To model the repeated measures, we will use GEE methods (Liang & Zeger 1986; Zeger & Liang 1986). When there is no additional random heterogeneity, 1−exp(*β*) represents the individual VE per exposure.

## 3. Assessing bias of our repeated measures model and the proportional hazards model under heterogeneous exposure intensities

In this section we show that VE based on one minus the hazard ratio of the first episode is a biased estimate of the VE per exposure (the individual VE or causal parameter of interest), when the assumption of proportional hazards is violated due to heterogeneous exposures to infection. We studied two scenarios of heterogeneous exposure: the first generated by the mixture of two Poisson distributions and the second by a continuous mixture of Poisson distributions. Using simulations, we also compared VE estimated through our repeated measures model to that estimated through proportional hazards under a heterogeneous and continuous intensity of exposure.

In the first exposure heterogeneity scenario, the population was assumed to be subdivided in two groups (*X*_*i*_=0/1). We allowed these two groups to receive exposures according to Poisson distributions, with means *λ*_1_ and *λ*_2,_ respectively. Under these assumptions, the interarrival times of each disease episode given the number of exposures received in the interval followed an exponential distribution, homogeneous across time, with mean $1/\lambda_{0}pg(\beta v_{i})$ when *X*_*i*_*=*0 and $1/\lambda_{1}pg(\beta v_{i})$ when *X*_*i*_*=*1, where *p* is the baseline probability that one exposure causes an event and *g*(*βv*_*i*_) represents the reduction in the probability of an event in one exposure contact conferred by the vaccine. The proportion of individuals in each of the two groups varied from 5 to 95%. Half of the subjects in each group were vaccinated with a vaccine with individual efficacy of 50%, to mimic a perfectly balanced randomized trial. The resulting distribution of exposure contacts was thus a mixture of two Poisson distributions with mean and variance equal to

$$\begin{array}{c} E(W_{ij})=\lambda =Pr(X_{i}=0)\lambda_{0}+Pr(X_{i}=1)\lambda_{1},\\ V(W_{ij})=Pr(X_{i}=0)[\lambda_{0}+{\left(\lambda_{0}-\lambda \right)}^{2}]+Pr(X_{i}=1)[\lambda_{1}+{\left(\lambda_{1}-\lambda \right)}^{2}]. \end{array}$$

**Figure 1:**
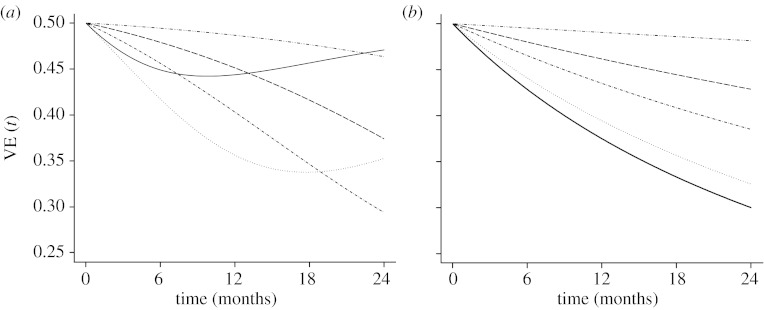
Population VE based on the hazard ratio comparing vaccinated with unvaccinated subjects as a function of time and individual VE of 50%. (*a*) Exposure was generated assuming a mixing distribution of two Poisson distributions with means *λ*_1_=12 and *λ*_2_=2, with the probability of occurrence of *λ*_2_ from 0.1 to 0.9, as specified. Triple dot-dashed line, 0.1; dashed line, 0.3; dot-dashed line, 0.5; dotted line, 0.7; solid line, 0.9. (*b*) Exposure was generated assuming a continuous mixture of Poisson distributions (negative binomial) with different means and variances (means and variances were chosen to mimic the mean and variance of the two point distribution). Triple dot-dashed line, mean=11 and variance=20; dashed line, mean=9 and variance=30; dot-dashed line, mean=7 and variance=32; dotted line, mean=5 and variance=26; solid line, mean=3 and variance=12.

When the exposure was based on a two-point distribution figure 1(*a*), the hazard was plotted based on

$$h(t_{i}|v_{i};\theta ,\lambda ,p)=\frac{\pi f(t_{i}|v_{i};p_{1},\theta ,\lambda )+(1-\pi )f(t_{i}|v_{i};p_{2},\theta ,\lambda )}{\pi S(t_{i}|v_{i};p_{1},\theta ,\lambda )+(1-\pi )S(t_{i}|v_{i};p_{2},\theta ,\lambda )}=\frac{\pi \lambda p_{1}\theta^{v_{i}}\;\text{exp}\left(-\lambda p_{1}\theta^{v_{i}}t_{i}\right)+(1-\pi )\lambda p_{2}\theta^{v_{i}}\;\text{exp}\left(-\lambda p_{2}\theta^{v_{i}}t_{i}\right)}{\pi \;\text{exp}\left(-\lambda p_{1}\theta^{v_{i}}t_{i}\right)+(1-\pi )\;\text{exp}\left(-\lambda p_{2}\theta^{v_{i}}t_{i}\right)}.$$

In the second exposure heterogeneity scenario, we assumed that half of the subjects were vaccinated with a vaccine of individual efficacy of 50%. The intensity of exposure for each subject (*λ*_*i*_) followed a gamma distribution with mean *λ* and variance *λ*/*γ*, leading to an overdispersed Poisson distribution for exposure with mean *λ* and variance *λϕ* (*ϕ*=(*γ*+1)/*γ*). The parameters *λ* and *γ* were chosen to match the mean and variance of the two group problems described above. The interarrival times of each disease episode given the number of exposures received in the interval, for subject *i*, were exponentially distributed with mean $1/\lambda_{i}pg(\beta v_{i})$.

When the exposure was based on a mixture of Poisson distributions (negative binomial, figure 1*b*), the hazard was plotted based on

$$h(t_{i}|v_{i};\theta ,\lambda ,p)=\frac{\int_{0}^{\infty }f(t_{i}|v_{i};p,\theta ,\lambda_{i})f(\lambda_{i})\;\text{d}\lambda_{i}}{\int_{0}^{\infty }S(t_{i}|v_{i};p,\theta ,\lambda_{i})f(\lambda_{i})\;\text{d}\lambda }=\frac{\int_{0}^{\infty }\theta^{v_{i}}p\lambda_{i}\;\text{exp}(-\theta^{v_{i}}p\lambda_{i}t_{i})[\gamma^{\lambda \gamma }/\Gamma (\lambda \gamma )]\lambda_{i}^{\lambda \gamma -1}\;\text{exp}(-\gamma \lambda_{i})\;\text{d}\lambda_{i}}{\int_{0}^{\infty }\text{exp}(-\theta^{v_{i}}p\lambda_{i}t_{i})[\gamma^{\lambda \gamma }/\Gamma (\lambda \gamma )]\lambda_{i}^{\lambda \gamma -1}\;\text{exp}(-\gamma \lambda_{i})\;\text{d}\lambda_{i}}=\frac{p\theta^{v_{i}}\lambda \gamma }{\gamma +p\theta^{v_{i}}t_{i}}.$$

Figure 1 plots the VE over time based on hazard ratios under these two scenarios. In a study with one year follow-up time, the population VE is always lower than the biological VE or VE per exposure (50%). After two years, VE based on hazard ratios could substantially underestimate the true (and constant over time) effect of the vaccine. The difference between the individual and the population vaccine efficacies is higher when most subjects have higher intensity of exposure (*λ*) or when the heterogeneity is large. The hazard of the mixing distribution generated by a binary intensity of exposure is similar to that generated by a continuous intensity of exposure, except when the mean of the mixing distribution is low. Similar numerical results were found by Schmoor & Schumacher (1997).

For both exposure scenarios, we generated datasets of 250 simulations with 1200 study subjects. Subjects were followed for 720 days and then censored (type I censoring only). For the repeated measures analysis, subjects were allowed to re-enter the study after each failure. We assigned vaccine to 50% of the study subjects and then sampled exposure using the corresponding distribution. The probability of infection per bite, *p*, was chosen to average a cumulative probability of disease over the two years in unvaccinated subjects of approximately 40% and VE was chosen to be 50%.

In all simulations, time was subdivided into intervals as if the subjects had been observed every 30 days. A binary outcome random variable was created, *D*_*ij*_, which was equal to 1 if the *i*th subject developed the outcome during the corresponding time period *j*. With these data, we fitted proportional hazards models for time to first event, frailty or random effect survival modelling time to first event (Gaussian frailty), our repeated measures model for all events using a complementary log–log link function and GEE, and a marginal multiple events survival model for continuous time to event, i.e. the Anderson–Gill model. All simulations and analyses were performed in Splus v. 8.0 (Insightful Corporation, Seattle). Estimation for the repeated measures model was implemented using the complementary log–log link and GEEs, assuming an independence working covariance matrix, using the *gee* function from the *correlatedData* library. Estimation for all survival models was implemented using the *coxph* function with the *cluster* function for the Anderson–Gill model and with the *frailty* function for the random effects survival model. Wald CIs were calculated based on the estimated standard errors.

**Figure 2:**
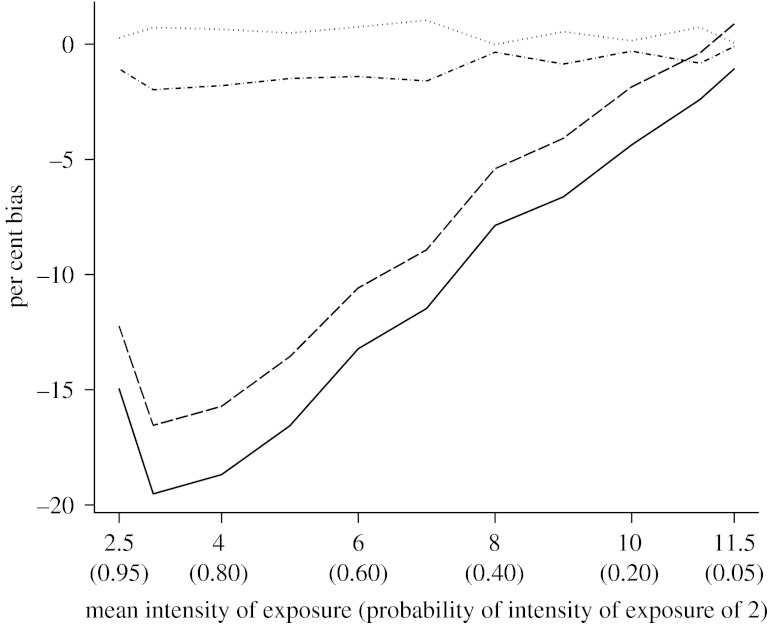
Comparison of the per cent bias in VE under heterogeneity of the intensity of exposure, as a function of the expected value of the mixing distribution of exposure, in 250 simulations each with a sample size of 1200 subjects. VE was estimated via modelling of time to first infection/disease (using proportional hazard and Gaussian frailty models), via modelling of time to all infection/disease (using Anderson–Gill model) and via a repeated measures model with a complementary log–log link and GEE approach. Exposure was generated assuming a continuous mixture of Poisson distributions with the specified mean intensity *λ* (and variance *ϕλ*). Dashed line, first event frailty model; dot-dashed line, repeated measures; dotted line, Anderson–Gill; solid line, first event proportional hazard.

When heterogeneity in the intensity of exposure varied continuously, the proportional hazards VE estimated without or with a random effect was biased. Our repeated measures estimator of individual efficacy performed substantially better and had negligible bias (figure 2). Results with our estimator and the Anderson–Gill approach were comparable. Discrepancies between our method and the Anderson–Gill approach are likely to be due to the discretization of time. While VE estimated by our method is based on the ratio of cumulative hazards over the specified time period, VE estimated by the Anderson–Gill method is based on the ratio of instantaneous hazards over the time period. Overall, our repeated measures model constituted a valid alternative to the Anderson–Gill approach, and in fact would be a more appropriate method to analyse data in which information about time to event is known in discrete time intervals (interval censoring). Moreover, although our estimator was based on discrete time to event, the half-widths of the 95% Wald CI of the estimator proposed here and that from the Anderson–Gill method were very similar. The difference was negligible in all simulation scenarios and at most 0.001.

## 4. Reanalysis of a malaria vaccine trial

We reanalyse the Brazilian trial of the SPf66 vaccine (Urdaneta *et al*. 1998) to compare the VE estimated through proportional hazards analysis (of first event only) with the VE estimated through our repeated measures estimator (of first and second events) implemented with a GEE approach. The SPf66 vaccine was expected only to protect vaccinees from disease, without affecting transmission (Graves *et al*. 1998; Graves & Gelband 2001).

In the Brazilian SPf66 vaccine trial, 58% of the study population had immigrated to the trial area in the two years prior to the trial, suggesting heterogeneous susceptibility to malaria among the study subjects. Although no mosquito surveys were performed in the trial area during the trial period, studies in nearby regions indicated that the intensity of exposure in the region ranged from 0.4 to 2.1 infected mosquito bites/person/night, depending on the season (Klein & Lima 1990; Urdaneta *et al*. 1996). A total of 800 individuals were randomized to vaccination (400) or placebo (400) and 572 (287 vaccinees versus 285 non-vaccinees) received three doses. As 32 of these individuals were lost to follow-up immediately after the third dose, the final analysis includes 540 study subjects. The study lasted 18 months after the third dose and recorded first and second malaria episodes.

Among the 540 subjects, 161 had one episode of falciparum malaria (the type of malaria with higher morbidity), and among those 44 presented with a second episode. The original trial analysed time to first infection/disease episode through life-table methods, with the hazard for each group estimated as the ratio of the number of cases at the end of the follow-up period to the person–time at risk. The trial reported a crude VE of 14.1% (95% CI of [−17.0, 36.9%]) for the first episode of malaria.

We performed a survival analysis for first episode using proportional hazards models, and a repeated measures analysis for first and second episodes, in monthly intervals, with GEEs using a working independence covariance assumption. In the repeated measures analysis, we assumed that the intensity of exposure (*λ*) was constant over time and, based on the previous entomological studies done in the area, equal to 30 infected bites/person/month. For this example, we chose three categorical covariates: vaccination; time living in the trial area; and age.

For each model, the estimated vaccine effect was low and did not reach statistical significance (table 1). Individuals who had lived in the area for more than two years had a lower susceptibility or probability of infection per exposure contact than those who were living in the area for two years or less. Neither the main effect of age nor its interaction with VE was significant in any analyses, indicating that baseline susceptibility and VE were relatively homogeneous across age groups. Adjusting for age or time living in the area did not appreciably change the point estimates of VE, suggesting no confounding due to these factors.

Overall, these results confirm the lack of VE found by Urdaneta *et al*. (1998). Currently, alternative delivery systems for the SPf66 vaccine are under investigation to improve the immunogenicity of the vaccine.

## 5. Discussion

The repeated measures model presented in this paper constitutes a practical way to estimate the individual VE or the VE conditional on exposure when the intensity of exposure is heterogeneous, even when the only knowledge about exposure relies on the estimated intensity of exposure from an environmental study. The estimator proposed here requires only a coarse estimate of the density of exposure. The repeated measures model offers a flexible and convenient way of handling heterogeneity in susceptibility and in the VE per exposure contact through covariates and random effects. Under unmeasured heterogeneity of the intensity of exposure in the population, our repeated measures model results in more accurate and more robust estimates of VE than the proportional hazards modelling of time to first event and offers an alternative to multiple events survival methods when the time interval of events rather than the exact event time is known.

When unmeasured heterogeneity leads to the failure of the proportional hazards assumption, survival analysis with frailty models for time to first event have been proposed to estimate the marginal or population-based VE under heterogeneity (Halloran *et al*. 1996; Longini & Halloran 1996). However, frailty single event models may also provide biased estimates of the individual VE, as shown here. As Hougaard (1991) remarks, single event frailty models are sensitive to the choice of the frailty distribution. Thus, many VE studies having recurrent events would be more appropriately analysed through multivariate methods, such as continuous time multivariate survival or repeated measures analysis. The methods assessed here are more robust than Poisson models that have been used by vaccine studies to handle multiple events (Alonso *et al*. 2005; Bejon *et al*. 2006). Poisson models rely on exponentially distributed interarrival times and, thus, yield only unbiased estimates of the individual VE when the assumption of proportional hazards is valid.

Although we specified our estimator for a Poisson distributed exposure, an alternative mixture of distributions for exposure, such as the negative binomial, could also be easily handled. In addition, an intensity of exposure varying over time and across subgroups of the population could be easily incorporated into the model. Assuming that exposures are Poisson distributed simplified the implementation of the model due to the resulting complementary log–log link function for the marginal probability of the outcome. Different motivations for complementary log–log models have been described (Collett 1991), including the discrete proportional hazards model. Therefore, our model to estimate VE can also be viewed as a multivariate version of the proportional hazards model, in which robust variances can be obtained through standard approaches such as GEEs. Frailties or random effects in susceptibility or in the log-transformed exposure can easily be incorporated within our longitudinal or repeated analysis framework.

We did not assess here the impact on the repeated measures estimator of having an exposure or susceptibility heterogeneously changing over time. A heterogeneous time-decreasing susceptibility could be the result of subjects' susceptibility decreasing conditionally on a recent event. Under these circumstances, our estimator could possibly be biased. However, including a covariate to represent recent event history, such as in a conditional Markov model, would probably correct most of this bias.

Since many vaccines may be given to prevent or delay diseases that recur, such as malaria, otitis media in pneumococcal infection or recurrent manifestations of perennial infections, such as the herpes or HIV virus, the proposed repeated measures estimator may be widely applicable. Our estimator is appropriate for the data collection mechanism of VE studies, since case detection at pre-specified time points often does not allow ascertainment of the exact failure time. Further developments of the general model proposed here could be made towards the estimation of individual VE when the infection/disease does not recur using a semi-parametric approach as suggested by van der Laan & Robins (2003). Extension to designs that collect detailed exposure information on a small validation set of participants and crude exposure information on all participants could be based on the semi-parametric methods of Golm *et al*. (1999). The model could also be extended to include estimation of ‘strain’-specific VE, as suggested by Gilbert *et al*. (1998), by classifying each event caused by a specific strain as a different outcome.

Given the results presented here, we recommend that primary analysis of vaccine trials with recurrent events should consider methods based on recurrent events, such as our repeated measures model, and not just the proportional hazards models for time to first event. It is probable that modelling time to first event yields biased estimates of individual VE. The VE estimated when modelling time to first event represents a parameter that varies with the transmission intensity in the trial site and the duration of the trial and, thus, does not allow vaccines to be judged. The repeated measures estimator proposed not only provides nearly unbiased estimates of the actual parameter of interest, individual VE, but also is an appropriate method to analyse data collected at discrete time intervals.

## Figures and Tables

**Table 1:** Point and CI estimates of VE and possible predictors of individual susceptibility, based on a survival analysis with proportional hazards model (using first episode only as outcome), and a repeated measures analysis with GEE of the Brazilian SPf66 malaria vaccine trial. Covariates include vaccination status, years living in the trial area prior to the trial and age.

variable	proportional hazards models		GEE models^a^	
	
	1^b^	2^b^	1^b^	2^b^
*P*(*D* | *W*=1, *V*=0) (%)	—	—	0.12 (0.09, 0.14)	0.11 (0.08, 0.14)
*VE* (%)	12.5 (−19.3, 35.8)	13.6 (−17.8, 36.6)	10.1 (−20.3, 32.8)	10.8 (−19.7, 33.4)
*RR*(time in area >2 versus ≤ 2 | *W*=1)	0.61 (0.44, 0.84)	0.61 (0.44, 0.85)	0.61 (0.44, 0.83)	0.61 (0.45, 0.84)
*RR*(age >20 versus age ≤ 20 | *W=*1)	—	1.17 (0.86, 1.60)	—	1.12 (0.83, 1.51)

a Standard errors of GEE models were calculated with robust variance estimators assuming a working independence covariance matrix.

b Models: model 1, ln(−ln(1−*Pr*{*d*_*ij*_|*v*_*i*_,*x*_*i*_}))=ln{*λ*=30}+*α*_0_+*β*_1_*v*_*i*_+α_2_*I*(time in area_*i*_>2); model 2, ln(−ln(1−*Pr*{*d*_*ij*_|*v*_*i*_,***x***_*i*_}))=ln{*λ*=30}+*α*_0_+*β*_1_*v*_*i*_+*α*_2_*I*(time in area_*i*_>2)+*α*_3_*I*(age_*i*_>20).
